# Disparities in time to treatment with oral antimyeloma medications

**DOI:** 10.1038/s41408-024-01128-1

**Published:** 2024-08-23

**Authors:** Hamlet Gasoyan, Faiz Anwer, Jeffrey D. Kovach, Nicholas J. Casacchia, Ming Wang, Jason Valent, Michael T. Halpern, Michael B. Rothberg

**Affiliations:** 1 Center for Value-Based Care Research, Department of Internal Medicine and Geriatrics, Primary Care Institute, Cleveland Clinic, Cleveland, OH USA; 2 Cleveland Clinic Lerner College of Medicine of Case Western Reserve University, Cleveland, OH USA; 3 Department of Hematology and Medical Oncology, Taussig Cancer Center, Cleveland Clinic, Cleveland, OH USA; 4 Department of Quantitative Health Sciences, Lerner Research Institute, Cleveland Clinic, Cleveland, OH USA; 5 Department of Population and Quantitative Health Sciences, Case Western Reserve University School of Medicine, Cleveland, OH USA; 6 Healthcare Delivery Research Program, National Cancer Institute, Bethesda, MD USA

**Keywords:** Health services, Myeloma

## Abstract

This retrospective cohort study used Taussig Cancer Center’s Myeloma Patient Registry to identify adults with multiple myeloma diagnosed between January 2017-December 2021. Electronic health records data captured time from diagnosis to initial prescription fill for oral antimyeloma medications and initiation of facility administered or oral antimyeloma treatment. We identified 720 patients with a mean age at diagnosis of 67 years ±11; 55% were male, 77% White, 22% Black, 1% other races, covered by private insurance (36%), traditional Medicare (29%), Medicare Advantage (25%), and Medicaid (8.3%). Over a third of patients (37%) resided in an area in the most disadvantaged area deprivation index (ADI) quartile. The median available follow-up was 765 days. Seventy-five percent of the cohort filled an oral antimyeloma medication prescription (excluding corticosteroids), with a median time to fill of 28 days (IQR, 15–61). In the multivariable Cox regression model, Black race (vs. White, adjusted hazard ratio [aHR], 0.61, 95% CI, 0.42–0.87), older age at diagnosis (aHR per 1 year, 0.97, 95% CI, 0.95–0.98), diagnosis during an inpatient admission (aHR, 0.63, 95% CI, 0.43–0.92), and estimated glomerular filtration rate ≤29 ml/min/1.73 m^2^ (vs. ≥60, aHR, 0.46, 95% CI, 0.29–0.73) were negatively associated with prescription fill for oral antimyeloma medication at 30 days, while insurance type and ADI were not significant predictors.

## Introduction

The development of orally administered antimyeloma medications, such as immunomodulatory drugs (IMiDs) lenalidomide and pomalidomide, is believed to have a major role in the improved survival of patients with multiple myeloma over the past two decades [1]. While oral formulations are more convenient, their high cost presents a formidable barrier to use [2–4]. Patient support programs can help pay for prescriptions, but they are challenging to navigate [5]. The multistep process of obtaining lenalidomide, including the risk evaluation and mitigation strategy (REMS) program requirements for patients and providers to complete mandatory surveys, lack of a functional phone line to complete the REMS program surveys, dispensing only via specialty pharmacies, courier delivery delays, and the time-consuming insurance prior authorization process, can also lead to delays in treatment initiation [6].

Disparities in the treatment initiation for multiple myeloma vary by demographic characteristics [4, 7–11] and insurance type [4, 10, 12]. For example, among patients who underwent autologous stem cell transplantation between 2000 and 2013 in a single center, referral for transplant was significantly delayed in Black individuals compared with White patients [7]. A National Cancer Database-based study of patients diagnosed between 2004 and 2015 found that initiation of treatment with any systemic antimyeloma treatment was delayed in females and Black patients (vs. White), while those were treated in a comprehensive community cancer program (vs. community cancer program) had lower odds of treatment initiation delay [10].

Less is known about disparities in the time to treatment initiation (TTI) with costly oral antimyeloma medications. Understanding how race and other social determinants of health affect delays in care is critical in planning health policy initiatives and patient navigation programs aimed at achieving equity [13]. The primary aim of this study was to examine the TTI with oral antimyeloma medications and examine whether there are disparities in the timing based on race and social determinants of health. We also captured TTI with either facility-administered or oral antimyeloma medication (excluding corticosteroids) and TTI of any treatment, including corticosteroids alone.

## Methods

### Study design and setting

This retrospective cohort study identified adult patients with multiple myeloma diagnosed between January 1, 2017, and December 31, 2021, using the Taussig Cancer Center’s Myeloma Patient Registry. Electronic health record (EHR) data from Cleveland Clinic’s Northeast Ohio sites, including linked Surescripts dispensation records [14], were used to capture the study variables and treatment received through December 31, 2022. Strengthening the Reporting of Observational Studies in Epidemiology guidelines were followed [15].

### Ethics approval and consent to participate

The study was approved by the Cleveland Clinic Institutional Review Board as minimal risk research using data collected for routine clinical practice, for which the requirement for informed consent was waived.

### Study participants

**Fig. 1:**
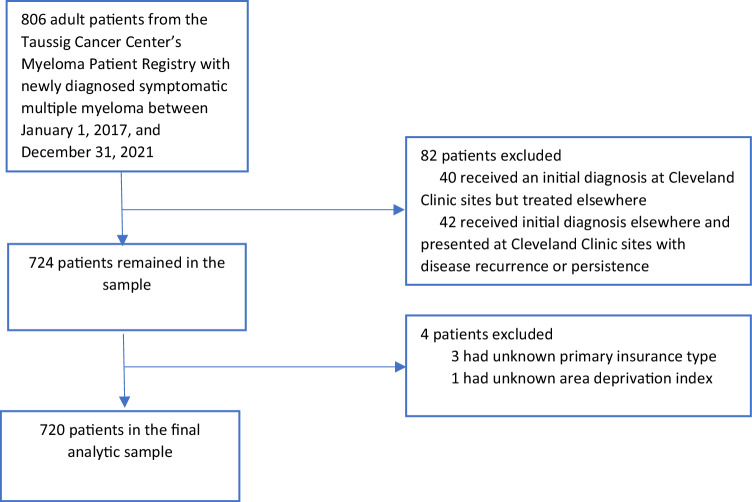
Identification of eligible patients for inclusion.

We identified adult (age 18 years and older) patients who were initially diagnosed with multiple myeloma (defined by International Classification of Disease [ICD]-O-3 code 9732/3 and histology description of active multiple myeloma in the Myeloma Patient Registry) between January 1, 2017, and December 31, 2021, and treated at Taussig Cancer Center—Cleveland Clinic’s specialized hub for cancer care—or Cleveland Clinic regional hospitals. Patients who received an initial diagnosis at Cleveland Clinic sites but were treated elsewhere and those who received an initial diagnosis elsewhere and presented at Cleveland Clinic sites with disease recurrence or persistence were excluded (Fig. 1).

### Study variables

The primary outcome was time from diagnosis to initial prescription fill for US Food and Drug Administration (FDA) approved oral medications with an indication for multiple myeloma [16]. The secondary outcome was time from the initial diagnosis to receipt of any FDA-approved oral or facility-administered medication with an indication for multiple myeloma [16]. For both of these outcomes, we did not consider the receipt of corticosteroids alone. However, given the clinical practice of initiating treatment with corticosteroids alone in some patients (due to delays in accessing specialty medications or when a patient chooses not to undergo chemotherapy), we also captured time from the diagnosis to receipt of any treatment, including corticosteroids.

Socio-demographic predictors included patients’ age at diagnosis, sex, race (based on patient self-report using fixed categories), primary insurance type, urbanicity of their primary residence based on Rural-Urban Commuting Area Codes [17], and Area Deprivation Index (ADI) based on Census Block Group [18]. Self-reported race was grouped into White, Black, and other categories. Primary insurance type, captured during the first visit at Cleveland Clinic on or after the initial diagnosis, were grouped into private insurance, traditional (fee for service) Medicare, Medicare Advantage, Medicaid, and self-pay/other payer categories. Urbanicity was classified into metropolitan, micropolitan, and small town or rural categories. ADI national rankings were grouped into quartiles, with higher scores reflecting greater disadvantage [18].

Clinical variables included the Charlson comorbidity index (CCI) [19], estimated glomerular filtration rate (eGFR) based on laboratory data captured closest to the initial diagnosis date (calculated using the 2021 CKD-EPI equation) [20], and Eastern Cooperative Oncology Group (ECOG) performance status. The latter represents the first available ECOG score in the EHR after initial diagnosis. Treatment facility was dichotomized between Taussig Cancer Center and Cleveland Clinic regional hospitals (including Avon Hospital, Fairview General Hospital, Hillcrest Hospital, Marymount Hospital, Medina Hospital, Mercy Medical Center, and South Pointe Hospital), where Cleveland Clinic oncologists provide the cancer treatment. Oncology practices at the regional hospitals follow a general oncology practice model, however, they have oncologists with expressed interest in multiple myeloma who receive preferential referrals of patients with multiple myeloma. Regional practices have similar resources compared to the Taussig Cancer Center. We also captured whether the initial diagnosis occurred during an inpatient stay since oral antimyeloma medications are not on the hospital formulary.

We also recorded the year of diagnosis and treated it as a categorical variable to account for the potential nonlinear relationships between the year of diagnosis and the expected increases in the uptake of the treatments, given that our study also included the pandemic period [21].

### Statistical analysis

Means and standard deviations were used to summarize normally distributed data and medians and interquartile ranges (IQRs) for data that were not normally distributed. Categorical variables were described using proportions. We calculated the proportions of patients who received either facility-administered or oral antimyeloma treatment, excluding corticosteroids (secondary outcome event), and those with a filled prescription for oral antimyeloma medications, excluding corticosteroids (primary outcome event) at 14 days and 30 days, respectively, based on the observed median time to these outcomes and clinical relevance [5]. Log-rank tests were used to compare the cumulative incidence curves of the primary and secondary outcome events by predictors of interest including age group, race, sex, primary insurance type, urbanicity, ADI quartile, and treatment facility. Multivariable Cox regression models were used to examine the associations of race, sex, age at diagnosis, primary insurance type, urbanicity, ADI quartile, treatment facility, and the primary and secondary outcome events (at the aforementioned time points), controlling for ECOG performance status and the year of diagnosis. The multivariable Cox model for the primary outcome was also adjusted for initial diagnosis during an inpatient stay and eGFR at diagnosis since oral antimyeloma medications are not on the hospital formulary and poor renal function may necessitate treatment delay with lenalidomide until renal function improves. The proportional hazards assumption in the multivariable Cox models were tested by examining scaled Schoenfeld residuals [22].

To better understand the drivers of race- and age-based disparities in the rates of initial prescription fill for oral antimyeloma medications at 30 days, we compared the unadjusted hazard ratios (HR) from the models that included only race or age as a predictor variable, respectively, with the adjusted HR (aHR) derived from the multivariable Cox model described above. We also captured the distribution of initial triplet or quadruplet regimens received among White and non-White patients. These regimens included common triplet (including bortezomib, lenalidomide, and dexamethasone; bortezomib, cyclophosphamide, and dexamethasone; carfilzomib, lenalidomide, and dexamethasone; daratumumab, lenalidomide, and dexamethasone) or quadruplet (including daratumumab, bortezomib, lenalidomide, and dexamethasone; and daratumumab, carfilzomib, lenalidomide, and dexamethasone) regimens available during the study period. We recorded a receipt of a triplet or quadruplet regimen if all its component medications were present in the EHR medication administration record and/or Surescripts dispensation records (for oral medications) within 35 days.

All statistical testing was two-tailed with an alpha of .05 used to determine statistical significance. All analyses were conducted in R, version 4.2.2 (R Foundation for Statistical Computing, Vienna, Austria).

## Results

We identified 720 patients with a mean age at diagnosis of 67 years ±11; 71% had ECOG status of 0–1, 55% were male, 77% White, 22% Black, and 1% other races, covered by private insurance (36%), traditional Medicare (29%), Medicare Advantage (25%), Medicaid (8.3%), and self-pay/other payor (1.9%). Over a third of patients (37%) resided in an area in the most disadvantaged ADI quartile; 83% resided in a metropolitan area. Most of the patients were treated at Taussig Cancer Center (84%) and the majority had an eGFR ≥60 ml/min/1.73 m^2^ at the time of diagnosis (61%). The median CCI was 2 (interquartile range [IQR], 0–5). The median available follow-up from diagnosis was 765 days (IQR, 401–1280) (Table 1).

**Table 1:** Characteristics of the study cohort, *N* = 720.

*Characteristic*	
**Sex, No (%)**	
Male	395 (55%)
Female	325 (45%)
**Race, No (%)**	
White	>553 (~77%)
Black	157 (22%)
Other^a^	<10 (~1%)
**Age at diagnosis, Mean (SD)**	67 (11)
**Charlson comorbidity index, Median (IQR)**	2 (0–5)
**ECOG performance status score, No (%)**	
0	214 (30%)
1	292 (41%)
2	126 (18%)
3	63 (8.8%)
4	15 (2.1%)
Unknown	10 (1.4%)
**Baseline eGFR (ml/min/1.73 m**^**2**^**), No (%)** ^b^	
≥60	436 (61%)
45–59	97 (13%)
30–44	70 (9.7%)
≤29	115 (16%)
Unknown	2 (0.3%)
**Year of initial diagnosis, No (%)**	
2017	142 (20%)
2018	133 (18%)
2019	143 (20%)
2020	142 (20%)
2021	160 (22%)
**Urbanicity of primary residence,**^c^ **No (%)**	
Metropolitan	597 (83%)
Micropolitan	75 (10%)
Small town or rural	48 (6.7%)
**Area Deprivation Index quartile,**^d^ **No (%)**	
1st quartile: 1–25	51 (7.1%)
2nd quartile: 26–50	168 (23%)
3rd quartile: 51–75	237 (33%)
4th quartile: 76–100	264 (37%)
**Primary insurance type, No (%)**	
Private insurance	260 (36%)
Traditional Medicare	206 (29%)
Medicare Advantage	180 (25%)
Medicaid	60 (8.3%)
Self-pay/other payor	14 (1.9%)
**Treatment facility**	
Taussig Cancer Center	608 (84%)
Regional hospitals	112 (16%)
**Initial diagnosis during hospital admission**	
No	564 (78%)
Yes	156 (22%)

*ECOG* Eastern Cooperative Oncology Group, *IQR* interquartile range, *SD* standard deviation.

^a^Cells with *n* < 10 were masked.

^b^Calculated using the 2021 CKD-EPI equation, based on laboratory data captured closest to the initial diagnosis date.

^c^Based on Rural-Urban Commuting Area Codes.

^d^The Area Deprivation Index national rankings range from 1 to 100, with higher scores reflecting greater disadvantage.

### Time to receipt of oral antimyeloma medications

During the study follow-up, 543 patients (75% of the cohort) filled a prescription for an oral antimyeloma medication (excluding corticosteroids). Among those with a filled prescription, the most common initial fill was for lenalidomide (93.7%), followed by cyclophosphamide (2.6%), pomalidomide (1.7%), ixazomib (1.5%), and thalidomide (0.6%). The median time to initial prescription fill was 28 days; with an IQR of 15–61, meaning that 25% of patients who received an oral antimyeloma medication had their prescriptions filled ≥2 months after their initial diagnosis. Among patients who received both oral and facility-administered antimyeloma medication (excluding corticosteroids), 274 (52%) filled their oral antimyeloma medication prescription on the same day or before receiving the facility-administered antimyeloma treatment.

**Fig. 2:**
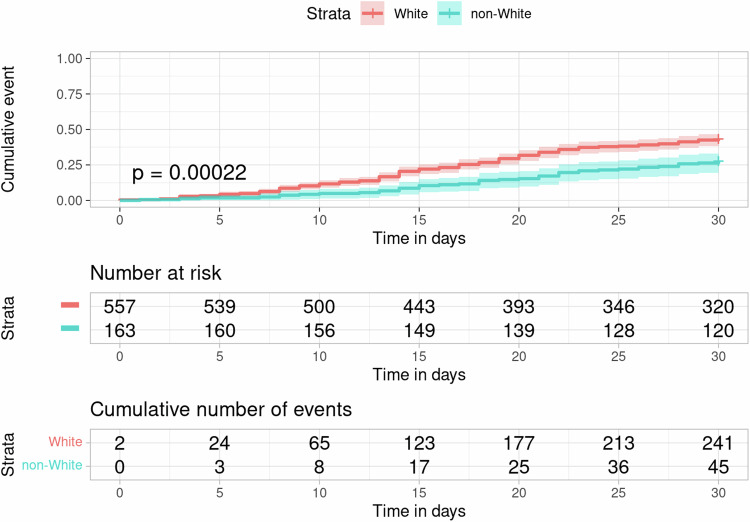
Cumulative incidence curves of oral anti-myeloma medication fill (excluding corticosteroids) by race, *N* = 720. Patients with self-reported other race and Black race were grouped into a non-White category to mask cells with *n* < 10. *P* value based on log-rank test. Shaded areas indicate 95% confidence intervals.

Overall, 286 patients (40% of the cohort) filled a prescription for oral antimyeloma medication (excluding corticosteroids) at 30 days after the initial diagnosis. The cumulative incidences of prescription fill for oral antimyeloma medication at 30 days differed by race, age group, baseline eGFR, treatment facility, and whether initial diagnosis occurred during hospital admission according to the log-rank tests (Table 2, Fig. 2). The cumulative incidence at 30 days was 43% (95% CI, 39–47%) in White individuals vs. 28% (95% CI, 21–36%) in Black patients; 65% (95% CI, 41–85%) in the ≤44 age group, 43% (95% CI, 32–55%) in the 45–54, 42% (95% CI, 35–49%) in the 55–64, 46% (95% CI, 40–52%) in the 65–74, and 24% (95% CI, 17–31%) in the ≥75 age group, respectively. The variation by insurance type approached but did not reach statistically different levels. The cumulative incidence at 30 days was 35% (95% CI, 28–42%) in patients with traditional Medicare, 38% (95% CI, 31–46%) with Medicare Advantage, 46% (95% CI, 40–52%) with private insurance, and 35% (95% CI, 23–48%) with Medicaid. In the unadjusted Cox regression models, Black race (vs. White, HR, 0.56, 95% CI, 0.41–0.78) and older age at diagnosis (HR per 1 year, 0.98, 95% CI, 0.97–0.99) were negatively associated with prescription fill for oral antimyeloma medication at 30 days, respectively. Other race was not a significant predictor in the model.

**Table 2:** Cumulative Incidence of anti-myeloma treatment initiation, by sociodemographic characteristics, *N* = 720.

Characteristic	Cumulative incidence of facility-administered or oral antimyeloma medication receipt at 14 days,^a^ No, (%), [95% CI]	Log-Rank P	Cumulative incidence of oral antimyeloma medication receipt at 30 days,^a^ No, (%) [95% CI]	Log-Rank P
**Sex**		0.2		0.3
Male	167 (42%), [37%, 47%]		151 (38%), [33%, 43%]	
Female	154 (47%), [42%, 53%]		135 (42%), [36%, 47%]	
**Race**		0.2		0.001
White	258 (46%), [42%, 51%]		241 (43%), [39%, 47%]	
Black	59 (38%), [30%, 46%]		44 (28%), [21%, 36%]	
Other ^b^	NR		NR	
**Age at diagnosis** (years)		0.5		<0.001
≤44 ^b^	NR		13 (65%), [41%, 85%]	
45–54	39 (53%), [41%, 64%]		32 (43%), [32%, 55%]	
55–64	90 (42%), [36%, 49%]		89 (42%), [35%, 49%]	
65–74	109 (45%), [38%, 51%]		112 (46%), [40%, 52%]	
≥75	75 (44%), [37%, 52%]		40 (24%), [17%, 31%]	
**Primary insurance**		0.6		0.06
Traditional Medicare	92 (45%), [38%, 52%]		72 (35%), [28%, 42%]	
Medicare Advantage	74 (41%), [34%, 49%]		69 (38%), [31%, 46%]	
Private insurance	124 (48%), [41%, 54%]		120 (46%), [40%, 52%]	
Medicaid	26 (43%), [31%, 57%]		21 (35%), [23%, 48%]	
Self-pay/other ^b^	NR		NR	
**Urbanicity** ^c^		0.4		0.7
Metropolitan	271 (45%), [41%, 49%]		235 (39%), [35%, 43%]	
Micropolitan	32 (43%), [31%, 55%]		33 (44%), [33%, 56%]	
Small town or rural	18 (38%), [24%, 53%]		18 (38%), [24%, 53%]	
**ADI quartile** ^d^		0.2		0.2
1st quartile: 1–25	25 (49%), [35%, 63%]		19 (37%), [24%, 52%]	
2nd quartile: 26–50	85 (51%), [43%, 58%]		77 (46%), [38%, 54%]	
3rd quartile: 51–75	105 (44%), [38%, 51%]		93 (39%), [33%, 46%]	
4th quartile: 76–100	106 (40%), [34%, 46%]		97 (37%), [31%, 43%]	
**Treatment facility**		0.4		<0.001
Taussig Cancer Center	276 (45%), [41%, 49%]		258 (42%), [38%, 46%]	
Regional hospitals	45 (40%), [31%, 50%]		28 (25%), [17%, 34%]	
**Diagnosis during hospital admission** ^e^	N/A			<0.001
No			250 (44%), [40%, 49%]	
Yes			36 (23%), [17%, 30%]	
**Baseline eGFR (ml/min/1.73 m**^**2**^**)** ^f^	N/A			<0.001
≥60			188 (43%), [38%, 48%]	
45–59			53 (55%), [44%, 65%]	
30–44			23 (33%), [22%, 45%]	
≤29			21 (18%), [12%, 27%]	

*ADI* Area Deprivation Index, *eGFR* estimated glomerular filtration rate, *N/A* not applicable, *NR* not reported.

^a^The time cut-offs were based on the observed median time to these outcomes and clinical relevance. For both outcomes, we did not consider the receipt of corticosteroids alone as antimyeloma treatment.

^b^Cells with *n* < 10 were masked.

^c^Based on Rural-Urban Commuting Area Codes.

^d^The Area Deprivation Index national rankings range from 1 to 100, with higher scores reflecting greater disadvantage.

^e^We compared cumulative incidence of oral antimyeloma medication receipt at 30 days based on whether the initial diagnosis occurred during an inpatient stay or not since these medications are not on the hospital formulary.

^f^Calculated using the 2021 CKD-EPI equation, based on laboratory data captured closest to the initial diagnosis date. We compared cumulative incidence of oral antimyeloma medication receipt at 30 days based on baseline eGFR since poor renal function may necessitate treatment delay with lenalidomide until renal function improves.

In the multivariable Cox regression model, Black race (vs. White, aHR, 0.61, 95% CI, 0.42–0.87), older age at diagnosis (aHR per 1 year, 0.97, 95% CI, 0.95–0.98), diagnosis during an inpatient admission (aHR, 0.63, 95% CI, 0.43–0.92), and eGFR ≤29 ml/min/1.73 m^2^ (vs. ≥60, aHR, 0.46, 95% CI, 0.29–0.73) were negatively associated with prescription fill for oral antimyeloma medication at 30 days. Other covariates in the model, including sex, primary insurance type, urbanicity, ADI quartile, treatment facility, ECOG performance status, and year of diagnosis were not associated with the primary outcome (Table 3). The model satisfied the proportional hazards assumption.

**Table 3:** Association of socio-demographic and clinical variables and oral antimyeloma medication receipt (excluding corticosteroids) at 30 days, *N* = 720.

Characteristic	Adjusted hazard ratio^a^	95% confidence interval^a^	*P* value^a^
**Sex**			
Male	reference		
Female	1.14	0.90–1.45	0.27
**Race**			
White	reference		
Black	0.61	0.42–0.87	0.007
Other	0.33	0.05–2.39	0.27
**Age at diagnosis** (per 1 year)	0.97	0.95–0.98	<0.001
**Primary insurance**			
Traditional Medicare	reference		
Medicare Advantage	1.14	0.81–1.61	0.46
Private insurance	0.84	0.57–1.24	0.38
Medicaid	0.71	0.40–1.26	0.25
Self-pay/other	0.39	0.14–1.11	0.08
**Urbanicity**^b^			
Metropolitan	reference		
Micropolitan	1.03	0.70–1.51	0.89
Small town or rural	0.74	0.45–1.23	0.25
**ADI quartile**^c^			
1st quartile: 1–25	reference		
2nd quartile: 26–50	1.04	0.62–1.73	0.88
3rd quartile: 51–75	0.87	0.53–1.45	0.60
4th quartile: 76–100	0.87	0.52–1.48	0.62
**Treatment facility**			
Taussig Cancer Center	reference		
Regional hospitals	0.89	0.58–1.37	0.61
**ECOG score**			
0	reference		
1	0.90	0.69–1.18	0.45
2–4	0.73	0.51–1.03	0.07
unknown	0.31	0.04–2.26	0.25
**Diagnosis during hospital admission** ^d^			
No	reference		
Yes	0.63	0.43–0.92	0.02
**Baseline eGFR (ml/min/1.73 m**^**2**^**)** ^e^			
≥60	reference		
45–59	1.82	1.32–2.51	<0.001
30–44	1.05	0.67–1.65	0.84
≤29	0.46	0.29–0.73	0.001
**Year of diagnosis**			
2017	reference		
2018	0.96	0.65–1.42	0.83
2019	1.38	0.94–2.02	0.10
2020	1.02	0.68–1.54	0.91
2021	1.20	0.83–1.73	0.33

*ADI* Area Deprivation Index.

^a^Based on multivariable Cox regression model.

^b^Based on Rural-Urban Commuting Area Codes.

^c^The Area Deprivation Index national rankings range from 1 to 100, with higher scores reflecting greater disadvantage.

^d^We adjusted the model for whether the initial diagnosis occurred during an inpatient stay or not since these medications are not on the hospital formulary.

^e^Calculated using the 2021 CKD-EPI equation, based on laboratory data captured closest to the initial diagnosis date. We adjusted the model for baseline eGFR since poor renal function may necessitate treatment delay with lenalidomide until renal function improves.

### Time to receipt of facility-administered or oral antimyeloma medications and any treatment

Overall, 666 patients (93% of the cohort) received treatment with facility-administered or oral antimyeloma medication (excluding corticosteroids) during the study follow-up. The distribution of initial triplet or quadruplet regimens did not significantly differ between White and non-White patients (eTable 1 in the Supplement). Among these patients, the median TTI of antimyeloma treatment was 15 days (IQR, 7–28).

At 14 days after initial diagnosis, 321 patients (45% of the cohort) received facility-administered or oral antimyeloma medication (excluding corticosteroids). The cumulative incidence of antimyeloma treatment initiation at 14 days did not significantly differ by race, age group, sex, primary insurance type, urbanicity, ADI quartile, or treatment facility (Table 2). In the multivariable Cox regression model, socio-demographic variables were not significantly associated with antimyeloma treatment initiation at 14 days (eTable 2 in the Supplement).

When considering receipt of any treatment, including corticosteroids alone, 695 patients (97% of the cohort) received such care, with a median time to treatment initiation of 9 days (IQR, 1–20). Almost six in ten (58%) patients in our study cohort initiated their treatment with corticosteroids alone.

## Discussion

In this study of 720 patients with newly diagnosed multiple myeloma, it took a median of 28 days from the time of diagnosis to receipt of an oral antimyeloma medication (excluding corticosteroids), but there was a wide interquartile range, stretching from 15 to 61 days. In the multivariable-adjusted model, Black race and older age at diagnosis were negatively associated with prescription fill for oral antimyeloma medication at 30 days, after controlling for relevant socio-demographic and clinical variables. Adjustment for sociodemographic and clinical variables attenuated the difference in HR for Black patients by only 0.05 (5% difference in relative risk) and further strengthened the negative association of age by 0.01 (1% difference in relative risk) per 1 year. This suggests an outstanding research gap related to the barriers affecting these populations that could be further examined via qualitative research approaches.

Our findings quantitatively measure the delays in treatment initiation with costly oral antimyeloma medications, identify independent predictors associated with no prescription fill for oral antimyeloma medication at 30 days, and highlight the need to focus on the barriers to timely treatment initiation in patients with multiple myeloma. Cleveland Clinic’s myeloma program quality metrics require that the time to treatment initiation for newly diagnosed multiple myeloma be not more than 10 days. The use of lenalidomide-containing combination induction therapies was a well-established standard of care during the study period, with lenalidomide intended to be taken at the onset of each treatment cycle [23]. Yet we found that half the patients experienced a time to fill for an initial oral antimyeloma medication of 28 days or more and one in four experienced a time to fill of 2 months or more. Our study cannot determine the reasons for these delays, but the TTI with oral antimyeloma medication was substantially longer than the time to initiate any treatment (including corticosteroids), which occurred at a median of 9 days. Other independent risk factors documented in our study were treatment initiation in an inpatient setting where these drugs are not on the hospital formulary and poor renal function at the time of diagnosis which could have necessitated treatment delay with lenalidomide until renal function improves. It is likely that the high cost of the medications led to the search for financial assistance grants by some patients. Other contributing factors to longer TTI with oral antimyeloma medications could have been the multistep process of REMS registration before a lenalidomide prescription can be ordered and delays related to the insurance precertification process. The race- and age-based disparities, which were present only for the oral antimyeloma medications suggest that the administrative burden, including for navigating the patient support programs to help pay for prescriptions, might delay treatment for these patients.

Fortunately, recent policy changes could help alleviate these barriers for Medicare beneficiaries. First, the Inflation Reduction Act of 2022 expanded eligibility for Medicare Part D low-income subsidy full benefits to up to 150% of the federal poverty level and eliminated the 5% coinsurance for catastrophic coverage starting in 2024 [24]. It will also cap out-of-pocket prescription drug spending at $2,000 (USD), effective in 2025 [24]. Second, the Center for Medicare and Medicaid Services has recently issued a final rule to address the administrative burden of obtaining prior authorization for patients covered by public insurance programs, by requiring third-party payors to instrument an automated process and make prior authorization decisions in shorter time frames and with more transparency [25]. The final rule would apply to healthcare services generally beginning January 2026 but oral prescription medications are not covered by this rule [25]. With the improved prior authorization processes via advanced technological interoperability, institutions could divert more resources from managing prior authorization-related administrative tasks to patient navigation programs. At the Cleveland Clinic, the multistep process of obtaining lenalidomide is facilitated by nurse coordinators, outpatient pharmacists, financial navigators, and social workers. Starting in 2023, the Cleveland Clinic also replaced an external specialty pharmacy with an internal specialty pharmacy to streamline the process.

Furthermore, the first generic version of lenalidomide is available and volume restrictions (established as part of the patent settlement process) are expected to be removed as of February 2026 [26]. This could lower the price for uninsured patients or those with Medicare (who are typically not eligible for manufacturer co-pay cards) and reduce the costs incurred by third-party payors. Finally, it should be also noted that out-of-pocket costs related to multiple myeloma treatment can vary considerably in privately insured patients. Particularly, Gasoyan and Rothberg recently reported that the median out-of-pocket cost during the first year after newly diagnosed multiple myeloma was $3711 (IQR, $1756 – $6466) in privately insured patients diagnosed in 2018; that number was as high as $5623 (IQR, $3517 – $7810) for patients with Consumer-Directed Health Plans or High-Deductible Health Plans [27]. As these plans become more prevalent among commercially insured individuals [28], policymakers should consider the barriers that such plans could impose on patients with multiple myeloma and other hematologic malignancies.

### Limitations

We used retrospective data from a myeloma patient registry and EHR, including Surescripts dispensation data, and included adult patients treated in a single large integrated health system in Ohio. Payer mix, insurance product type, and other clinical or sociodemographic factors may vary by facility and geographic location. Furthermore, most of the study participants were treated at a specialized cancer center, which may limit the generalizability of our findings. A limited number of patients might have received oral antimyeloma medications as part of clinical trials without being captured in our data. Finally, while we captured the primary insurance type, we were not able to document the insurance plan benefit details such as out-of-pocket cost maximums, or whether our study participants used patient support programs to help pay for prescriptions. The particular strengths of our study were its large sample spanning multiple years, our ability to confirm the initial diagnosis date via the Myeloma Patient Registry, and combine clinical data (such as performance status and eGFR data), captured from the EHR, with documented prescription fills, from a validated data source [14].

## Conclusions

This study assessed factors associated with delayed treatment initiation with oral antimyeloma medications in patients with newly diagnosed multiple myeloma. We found considerable discrepancies between the timing of any treatment initiation and oral antimyeloma medication fill. Black and older patients were less likely to get an oral antimyeloma medication at 30 days. Our findings highlight the need to focus on the barriers to simultaneous initiation of all components of guideline-recommended multidrug induction regimens for multiple myeloma and identify patient populations who might be at higher risk of delayed treatment initiation with oral antimyeloma medications.

### Supplementary information

Supplementary Appendix

## Data Availability

The datasets generated during and/or analyzed during the current study are not publicly available to protect patient confidentiality but are available from the corresponding author upon reasonable request.
