# Supplementary material for: Disparities in time to treatment with oral antimyeloma medications
Source: Blood Cancer J. 2024 Aug 23;14(1):142. doi: 10.1038/s41408-024-01128-1 (PMC11343868; doi:10.1038/s41408-024-01128-1)
Supplement: Supplementary file 1 — Supplementary Appendix [file 41408_2024_1128_MOESM1_ESM.docx]

# Supplementary Appendix

Disparities in Time to Treatment with Oral Antimyeloma Medications

**eTable 1. Distribution of initial triplet and quadruplet regimens among White and non-White patients, N=720.**

|  | **White,** n=557 | **non-White**, n=163 | **P value** *^1^* |
| --- | --- | --- | --- |
| **Regimen, No (%)** |  |  | 0.8 |
| VRd ^2^ | 202 (36%) | 64 (39%) |  |
| CyBorD ^2^ | 97 (17%) | 33 (20%) |  |
| KRd ^2^ | 16 (2.9%) | 5 (3.1%) |  |
| Dara-VRd ^2^ | 12 (2.2%) | 1 (0.6%) |  |
| DRd ^2^ | 8 (1.4%) | 2 (1.2%) |  |
| Dara-KRd ^2^ | 1 (0.2%) | 0 (0%) |  |
| No triplet or quadruplet regimen | 221 (40%) | 58 (36%) |  |

*Abbreviations: VRd, bortezomib, lenalidomide, and dexamethasone; CyBorD, bortezomib, cyclophosphamide, and dexamethasone; KRd, carfilzomib, lenalidomide, and dexamethasone; DRd, daratumumab, lenalidomide, and dexamethasone; Dara-VRd, daratumumab, bortezomib, lenalidomide, and dexamethasone; Dara-KRd, daratumumab, carfilzomib, lenalidomide, and dexamethasone. ^1^ Based on Fisher’s exact test. ^2^ We recorded a receipt of a triplet or quadruplet regimen if all its component medications were present in the EHR medication administration record and/or Surescripts dispensation records (for oral medications) within a 35-day period.*

**eTable 2. Association of socio-demographic and clinical variables and receipt of facility administered or oral antimyeloma medication (excluding corticosteroids) at 14 days, N=720.**

| **Characteristic** | **Adjusted hazard ratio**^1^ | **95% confidence interval**^1^ | **P value**^1^ |
| --- | --- | --- | --- |
| **Sex** |  |  |  |
| Male | reference |  |  |
| Female | 1.16 | 0.93-1.44 | 0.20 |
| **Race** |  |  |  |
| White | reference |  |  |
| Black | 0.86 | 0.62-1.18 | 0.35 |
| Other | 1.62 | 0.59-4.41 | 0.35 |
| **Age at diagnosis** (per 1 year) | 1.00 | 0.98-1.01 | 0.62 |
| **Primary insurance** |  |  |  |
| Traditional Medicare | reference |  |  |
| Medicare Advantage | 0.87 | 0.63-1.19 | 0.37 |
| Private insurance | 1.07 | 0.76-1.52 | 0.69 |
| Medicaid | 1.03 | 0.62-1.73 | 0.90 |
| Self-pay/other | 0.68 | 0.27-1.72 | 0.42 |
| **Urbanicity**^2^ |  |  |  |
| Metropolitan | reference |  |  |
| Micropolitan | 0.86 | 0.59-1.26 | 0.45 |
| Small town or rural | 0.78 | 0.48-1.28 | 0.33 |
| **ADI quartile**^3^ |  |  |  |
| 1st quartile: 1–25 | reference |  |  |
| 2nd quartile: 26–50 | 1.03 | 0.65-1.62 | 0.91 |
| 3rd quartile: 51–75 | 0.88 | 0.56-1.38 | 0.58 |
| 4th quartile: 76–100 | 0.76 | 0.48-1.21 | 0.25 |
| **Treatment facility** |  |  |  |
| Taussig Cancer Center | reference |  |  |
| Regional hospitals | 0.81 | 0.58-1.15 | 0.24 |
| **Year of diagnosis** |  |  |  |
| 2017 | reference |  |  |
| 2018 | 0.95 | 0.67-1.36 | 0.79 |
| 2019 | 0.94 | 0.66-1.35 | 0.76 |
| 2020 | 0.95 | 0.66-1.36 | 0.77 |
| 2021 | 0.95 | 0.67-1.33 | 0.75 |

*Abbreviations: ADI, Area Deprivation Index. ^1^Based on multivariable Cox regression model. We also controlled for ECOG score by allowing separate baseline hazard functions for each of its levels (0, 1, 2-4, unknown) and for hazard ratios to vary across these groups. Inclusion of ECOG score as a predictor variable (as opposed to stratifying by ECOG score) would have violated the proportional hazards assumption in the model. ^2^Based on Rural-Urban Commuting Area Codes, ^3^The Area Deprivation Index national rankings range from 1 to 100, with higher scores reflecting greater disadvantage.*
